# Application of Methionine Increases the Germination Rate of Maize Seeds by Triggering Multiple Phenylpropanoid Biosynthetic Genes at Transcript Levels

**DOI:** 10.3390/plants12223802

**Published:** 2023-11-08

**Authors:** Ying Ren, Fengyuan Shen, Ji’an Liu, Wenguang Liang, Chunyi Zhang, Tong Lian, Ling Jiang

**Affiliations:** 1 Biotechnology Research Institute, Chinese Academy of Agricultural Science, Beijing 100081, China; ryry0612@163.com (Y.R.); shenfengyuan@126.com (F.S.); liujian2021123@sina.cn (J.L.); sby184@163.com (W.L.); zhangchunyi@caas.cn (C.Z.); 2 Sanya Institute, Hainan Academy of Agricultural Sciences, Sanya 572000, China; 3 Hainan Yazhou Bay Seed Laboratory, Sanya 572000, China

**Keywords:** methionine, germination, transcriptomic analysis, maize

## Abstract

Methionine is an essential amino acid that initiates protein synthesis and serves as a substrate for various chemical reactions. Methionine metabolism plays an important role in *Arabidopsis* seed germination, but how methionine works in seed germination of maize has not been elucidated. We compared the changes in germination rate, the contents of methionine and folates, and transcriptional levels using transcriptome analysis under water or exogenous methionine treatment. The results indicate that the application of methionine increases seed germination rate (95% versus 70%), leading to significant differences in the content of methionine at 36 h, which brought the rapid increase forward by 12 h in the embryo and endosperm. Transcriptome analysis shows that methionine mainly affects the proliferation and differentiation of cells in the embryo, and the degradation of storage substances and signal transduction in the endosperm. In particular, multiple phenylpropanoid biosynthetic genes were triggered upon methionine treatment during germination. These results provide a theoretical foundation for promoting maize seed germination and serve as a valuable theoretical resource for seed priming strategies.

## 1. Introduction

Germination represents the pivotal stage in the life cycle of seed plants, and is defined as the process from the initial absorption of seed water to the eventual penetration of the radicle through the surrounding tissues and the seed coat [1]. Seed germination can be categorized into three stages. In the initial stage, dry seeds swiftly absorb water and undergo expansion—a physical process also observed in non-viable seeds. At this point, the seed cells restore their original structure and function, enzyme proteins resume their activity, certain genes in the cells begin to be expressed, and storage substances in the endosperm begin to decompose. In the second stage, various metabolites and signal transduction processes are activated, including hormone accumulation, DNA synthesis and repair, protein translation, post-translational modifications, and the regulation of sulfur-containing amino acid metabolism during seed germination. Storage substances gradually transform into simple soluble compounds. Amino acids, glucose, glycerol, and fatty acids are further converted into transportable compounds such as polyphthalamine and sucrose. When favorable environmental conditions occur, these changes enable triggered seeds to develop toward the third stage, which is the radicle protrusion, to complete germination and enter the seedling stage [2].

The germination of seeds results from the interaction between the external environment and the internal factors of the seeds themselves. The known factors that affect the germination of corn seeds can be divided into two categories: one comprises the external ecological conditions such as water, temperature, light, soil conditions, sowing depth, and other environmental factors; the other comprises the genetic characteristics of the seeds themselves. This includes whether the seed itself has sufficient accumulation of storage substances such as protein and starch, as well as the ability to use these storage substances and hormones to receive external signals to initiate biochemical reactions in life activities [2]. The convergence of these two factors ultimately governs seed germination.

Genetic characteristics of the seeds that are important for germination have been well studied. Plant hormones play a pivotal role in seed germination. Two well-established plant hormones, abscisic acid (ABA) and gibberellin (GA), exert significant influences on this crucial phase. Numerous studies have highlighted the importance of ABA catabolism in altering seed dormancy. The breakdown of ABA, achieved through a sequence of hydroxylation and conjugation reactions, acts as a catalyst for seed germination. Simultaneously, key components in the ABA signaling pathway, such as the protein kinase SnRK2s (sucrose non-fermenting-1-related protein kinase 2s) and downstream transcription factors ABI3/4/5 (abscisic-acid-insensitive transcription factors), can be directly or indirectly activated or inhibited by various factors, further promoting seed germination [3,4]. GA induces seed germination and promote the elongation of hypocotyls and stems. The genes involved in GA biosynthesis and transduction are important for germination, such as SEMIDWARF1 (SD1) and GIBBERELLIN INSENSIVE DWARF1 (GID1) [5,6]. Upstream of the GA synthesis and degradation pathway, V-myb avian myeloplastosis viral oncogene homolog (MYB) transcription factors are also involved in the regulation of maize seed germination. Maize *ZmMYB59* can activate the expression of *GA2Ox-3* and *GA2Ox-10* encoding gibberellin 2 oxidase by binding to the ATCCACC element in plants, leading to the conversion of active GA into an inactive form, thereby reducing the endogenous GA content in plants and inhibiting maize seed germination [7]. Moreover, it is important to note that plant hormones do not only act alone; many of them also have antagonistic or synergistic effects with each other to better assist plant growth and development, such as the interaction relationship between jasmonic acid (JA), ABA, and GA. GA can affect the content of JA through RGA-LIKE 2 (RGL2), a member of the DELLA protein, a major inhibitor of *Arabidopsis* germination, and ABA can also promote JA synthesis through the SAPK10–bZIP72–AOC pathway. JA can activate ABA signaling by degrading the JAZ protein (JASMONATE ZIM DOMAIN, an inhibitor of JA response), releasing ABA-insensitive transcription factors (ABI3 and ABI5), thereby negatively regulating seed germination [8,9]. Indeed, various other plant hormones, including auxin, cytokinin, strogolactone, brassinosteroids, and ethylene exert significant influences on plant germination [10,11,12,13,14]. Additionally, signaling substances such as reactive oxygen species (ROS), nitric oxide, and sulfur dioxide have an impact on seed germination by affecting GA biosynthesis and embryonic root development [15,16,17]. Furthermore, phenylpropanoid biosynthesis, including phenylalanine metabolism, also are important for the seed germination [18,19].

In previous investigations, methionine metabolism has emerged as a pivotal player in seed germination in *Arabidopsis*. The application of exogenous methionine can activate the *Arabidopsis* glutamate receptor homolog AtGLR 3.5 Ca^2+^ channel, promote the Ca^2+^ influx, increase intracellular Ca^2+^ concentration, inhibit the expression of ABI4, reduce seed sensitivity to ABA, promote seed germination, and ultimately increase seed germination rate [20]. The protein level of methionine synthase was significantly increased in the proteome of *Arabidopsis* seeds before the embryonic roots broke through the seed coat after 24 h of imbibition. After 48 h of imbibition, the content of S-adenosylmethionine (SAM) synthetase (SAMS) was significantly increased after the embryo root broke through the seed coat [21,22]. The impact of methionine content on maize germination at the molecular level remains unclear. To gain insight into how methionine participates in maize seed germination, the exogenous application of methionine was used on maize B73 seeds during germination, germination rate, the contents of methionine and folate, transcriptional levels using transcriptome analysis, and protein–protein interaction were analyzed.

## 2. Results

### 2.1. Effect of the Exogenous Methionine Application on Maize Seed Germination

**Figure 1:**
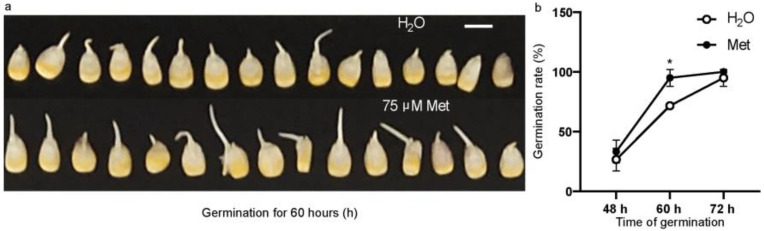
Germination rate of maize seeds after exogenous methionine application. (**a**) A photo shows seed germination phenotypes with water or 75 μM methionine at 60 h, and bar = 1 cm; (**b**) seed germination rate of water (empty cycles) or after the application of 75 μM methionine (black dots) at different time points of germination. *, *p* < 0.05.

In order to explore the role of methionine in the germination process, inbred line B73 seeds were soaked in 75 μM methionine as a treatment and water as a control. The effect of exogenous methionine treatment on corn seed germination was studied by observing the changes in the germination time and germination rate. At 60 h of germination, it was seen that almost all seeds soaked in 75 μM methionine had germinated, while some seeds in water had not germinated (Figure 1a). During the first 36 h, corn seeds were in the first and second stages of germination, absorbing water and swelling, and the embryonic roots did not protrude from the seed coat. During the process of germination from 36 to 48 h, the embryonic roots began to break through the seed coat, so the germination rates at 48 h, 60 h, and 72 h were compared. The results show that approximately 25% of the B73 seeds had germinated after 48 h of water treatment, and approximately 33% had germinated after treatment with methionine. At 60 h, seeds treated with 75 μM methionine had almost fully germinated (with a germination rate of 95 ± 5%), while the control group treated with water had a germination rate of approximately 70% (Figure 1b). At 72 h, almost all seeds treated with water and methionine had germinated. This result shows that methionine increases the germination rate of corn seeds under this concentration.

### 2.2. Effect of the Application of Methionine on the Contents of Free Methionine and 5-Methyl-tetrahydrofolate (5-M-THF) in Seeds

**Figure 2:**
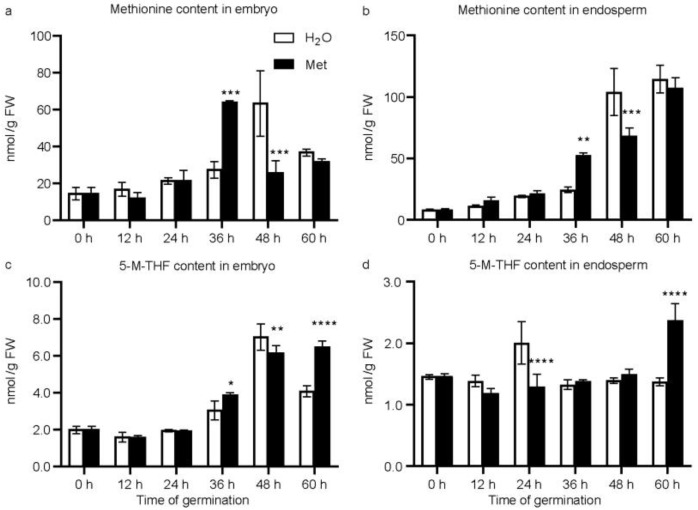
Changes in the contents of methionine (Met) and 5-methyl-tetrahydrofolate (5-M-THF) in the embryo and endosperm during germination upon the treatment of water (H_2_O, white bars) or Met (black bars). (**a**) Met contents in the embryo; (**b**) Met contents in the endosperm; (**c**) 5-M-THF contents in the embryo; (**d**) 5-M-THF contents in the endosperm. *, *p* < 0.05; **, *p* < 0.01; ***, *p* < 0.001; ****, *p* < 0.0001.

The main pathway of methionine synthesis is the generation of methionine and tetrahydrofolate using 5-M-THF and homocysteine as substrates [23]. Folates are coenzymes mediating the transfer of one carbon unit to participate in the synthesis of purines and pyrimidines, which is closely related to cell division [24]. After the application of methionine, free methionine and folates were extracted from the embryo and endosperm of maize seeds at 0 h, 12 h, 24 h, 36 h, 48 h, and 60 h, respectively, during the germination process. In the water-treated group, the methionine content increased sharply from 36 to 48 h, and reached its peak (63.61 ± 17.24 nmol/g FW) at 48 h in the embryo. It was approximately 4.29-fold that of the initial state at 0 h (14.83 ± 2.81 nmol/g FW). When treated with methionine, the overall trend of the methionine content in the embryo was similar to that of water germination, but the time at which the methionine content reached its peak was advanced from 48 h to 36 h. The methionine content increased rapidly at 36 h (64.22 ± 0.60 nmol/g FW), which was approximately 4.33-fold of the initial value, and it then decreased (Figure 2a). In the endosperm, under water germination, the content of methionine continuously increased during the period of 0–60 h. During the first 36 h, the content of methionine slowly increased; then, at 48 h (103.94 ± 19.08 nmol/g FW), the content of methionine increased rapidly, being approximately 12.08-fold of the initial state at 0 h (8.60 ± 0.76 nmol/g FW). At 60 h, the content of methionine (114.53 ± 11.39 nmol/g FW) was still increasing. After the application of methionine, the overall trend of the methionine content in the endosperm was similar to that under water germination, and the content of methionine started to increased rapidly at 36 h, and the methionine content (52.52 ± 1.93 nmol/g FW) was 2.14-fold of that in the water treatment (24.51 ± 2.18 nmol/g FW) at the same time point (Figure 2b). Overall, the embryo and endosperm synthesized methionine de novo during the water absorption, and germination process and significant differences in the content of seed methionine at 36 h, which brought the rapid increase forward by 12 h in the embryo and endosperm, were observed.

Compared with the pattern of methionine, in the embryo, 5-M-THF did not change significantly from 0 to 24 h of germination with or without the addition of methionine. It began to increase rapidly at 36 h (3.08 ± 00.46 nmol/g FW), and the 5-M-THF content in the water treatment reached its peak at 48 h (7.06 ± 0.66 nmol/g FW); it then decreased at 60 h (4.09 ± 0.27 nmol/g FW). The 5-M-THF content in the methionine treatment continued to increase rapidly after 36 h (3.91 ± 0.08 nmol/g FW); in the later stage, the content at 60 h (6.50 ± 0.30 nmol/g FW) was significantly higher than that in the water treatment (Figure 2c). Conversely, no obvious changes were observed in the endosperm upon methionine treatment (Figure 2d).

### 2.3. Transcriptome Analysis during Maize Germination

To conduct a comprehensive analysis of the impact of exogenous methionine treatment on the germination process of corn seeds, embryos and endosperms from B73 seeds treated with water and methionine at six time points of germination (0 h, 12 h, 24 h, 36 h, 48 h, and 60 h) were isolated for transcriptome sequencing. A total of 66 samples were collected for RNA-sequencing, and the sequencing data of 640.20 Gb clean reads were obtained with the GC content of each sample was around 50–60%. The Q30 averaged over 88.38% and Q20 averaged over 95%, indicating that the quality of the sequencing data was good for the analysis requirements. Using the Hisat2 perform, the sequence alignment of clean reads with the maize reference genome was performed (Zea_mays_AGPv4.37, ftp://ftp.ensemblgenomes.org/pub/plants/release-37/fasta/zea_mays/dna/Zea_mays.AGPv4.dna.toplevel.fa.gz, accessed on 30 August 2017), and the alignment efficiency ranged from 94.56% to 98.53%. The only comparison rate to the reference genome was between 88–95% (Tables S1 and S2). Using the selected reference genome sequence, newly discovered genes were sequenced and compared against major databases, resulting in the identification of a total of 13,965 new genes by comparing them with existing genome annotation information. These new genes were subsequently annotated using various databases, yielding the number of newly annotated genes in each database (Table S3).

### 2.4. Differentially Expressed Genes (DEGs) Analysis

**Figure 3:**
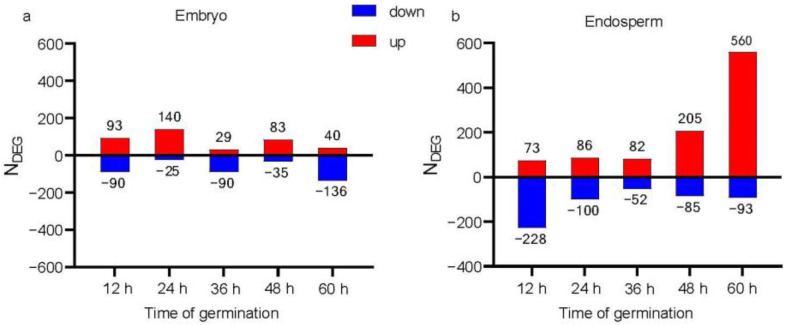
The numbers of differentially expressed genes (DEGs) in the embryo and endosperm under methionine treatment compared with water treatment. (**a**) The number of DEGs in the embryo under methionine treatment; (**b**) the number of DEGs in the endosperm under methionine treatment.

To identify genes that respond to methionine treatment during maize seed germination, DEGs were screened using log2 (FC) ≥ 1.5 as the standard. Water treatment was used as the control, and differences were compared between methionine treatment and water treatment at the same time point. In the embryo at 12 h, 183 DEGs were detected, of which 93 were upregulated and 90 were downregulated. At 24 h, there were 165 DEGs, with 140 upregulated and 25 downregulated. A total of 119 DEGs were detected at 36 h, of which 29 were upregulated and 90 were downregulated. At 48 h, 118 DEGs were detected, of which 83 were upregulated and 35 were downregulated. At 60 h, there were 176 DEGs, of which 40 were upregulated and 136 were downregulated (Figure 3a). In the embryo, there were more upregulated genes at 12, 24, and 48 h than downregulated genes, and there were more downregulated genes at 36 and 60 h. This result indicates that the application of methionine promotes and inhibits gene transcription levels in a 12-h rhythm in the embryo. In the endosperm, the number of upregulated genes increased over the time, especially at 48 and 60 h, and the number of downregulated genes decreased notably in the first 36 h and then increased (Figure 3b). There were more DEGs in the endosperm than in the embryo. Compared with the embryo, a 12-h rhythm was not observed in the endosperm. In the endosperm, most downregulated genes were expressed at 12 h, and the number of DEGs at 60 h accounted for half of the total number of DEGs. Moreover, the majority of DEGs at 60 h were upregulated. This result indicates that in the late stage of treatment in the endosperm, more genes’ transcription levels increase in response to methionine treatment, when seeds treated with 75 μM methionine have almost fully germinated (Figure 1 and Figure 3).

### 2.5. Gene Ontology (GO) Analysis of the DEGs

**Figure 4:**
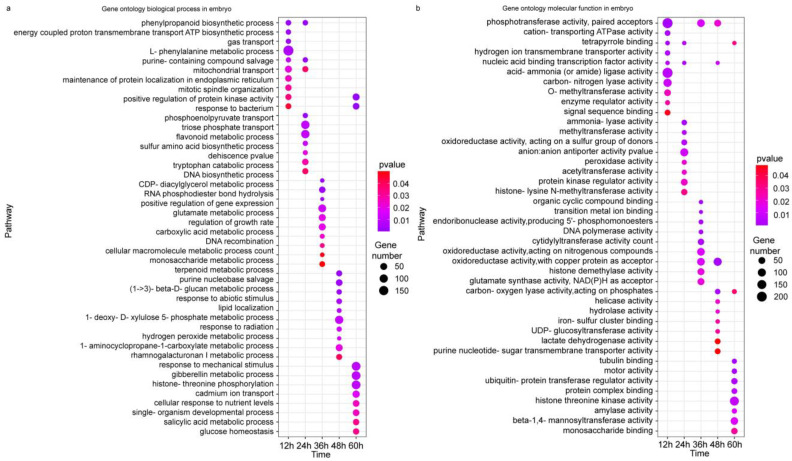
Gene Ontology (GO) analysis of transcriptional differences in the embryo during the germination of maize seeds treated with exogenous methionine. (**a**): Top 20 GO biological process enrichments from different time points; (**b**): top 20 GO molecular function enrichments from different time points. The bubble color indicates the *p* value. The size of the dots indicates how many differential genes are present in each pathway.

**Figure 5:**
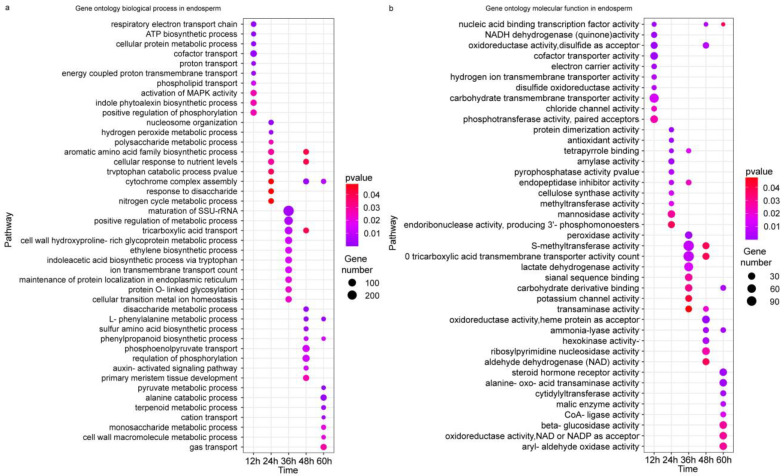
Gene Ontology (GO) analysis of transcriptional differences in the endosperm during the germination of maize seeds treated with exogenous methionine. (**a**): Top 20 GO biological process enrichments from different time points; (**b**): top 20 GO molecular function enrichments from different time points. The bubble color indicates the *p* value. The size of the dots indicates how many differential genes are present in each pathway.

GO analysis reveals more details of the differences at the transcriptional level upon the application of methionine in maize embryos and endosperms during germination. In the early stage of embryo development (12–24 h), there were three biological processes (phenylpropanoid biosynthetic process, L-phenylalanine metabolic process, purine-containing compound salvage) that were exhibited repeatably, and they all appeared at 12 and 24 h. At 24 h, a number of genes involved phosphoenolpyruvate transport and triose phosphate transport were enriched. At 36 h, most of the genes were involved in the positive regulation of gene expression, the glutamate metabolic process, and regulation of growth rates. At 48 h, glycolysis (1-deoxy-D-xylulose 5-phosphate metabolic process), purine nucleobase salvage, and ethylene biosynthesis (1-aminocyclopropane-1-carboxylate metabolic process) were the most enriched. At 60 h, the gibberellin metabolic process, histone–threonine phosphorylation, cadmium ion transport, and the salicylic acid metabolic process were enriched (Figure 4a). There were three molecular functions (phosphotransferase activity, tetrapyrrole binding, and nucleic acid binding transcription factor activity) detected at three time points, and they all appeared at 12 h. Oxidoreductase activity acting on different molecules was detected at different points (sulfur, 24 h; nitrogenous compounds and copper protein, 36 h), indicating more active redox processes upon methionine treatment. The same was observed for lyase activity, such as carbon–nitrogen lyase at 12 h and carbon–oxygen lyase at 36 h. At 60 h, more genes were enriched in histone–threonine kinase activity (Figure 4b). Differing from the biological processes enriched in the embryo, the enriched biological processes in the endosperm were mainly involved in molecule transport (cofactors, protons, energy-coupled proton transmembrane, phospholipids at 12 h; tricarboxylic acid at 36 h; phosphoenolpyruvate at 48 h; cations and gas at 60 h), hormone transduction (indole phytoalexin at 12 h; ethylene and indoleacetic acid at 36 h; auxin at 48 h), positive regulation (phosphorylation at 12 h and metabolic process at 36 h), and all types of metabolic processes at different time points, including hydrogen peroxide, polysaccharide, cell wall hydroxyproline-rich glycoprotein, disaccharide, L-phenylalanine, sulfur amino acids, phenylpropanoids, pyruvate, terpenoid, monosaccharides, and cell wall macromolecules. The phenylpropane synthesis pathway, phenylalanine metabolism pathway, and cytochrome complex pathway were repeatedly enriched at 48 and 60 h (Figure 5a). At 12 h, the molecular functions enriched in the endosperm were almost the same as those in the embryo, but oxidoreductase activity using disulfide as an acceptor and disulfide oxidoreductase activity were not found in the embryo. S-Methyltransferase and tricarboxylic acid transmembrane transporter activity were most enriched during germination, and steroid hormone receptor activity was also enriched (Figure 5b). In the mid-stage of germination, various hormone pathways and hydrolase activity were enriched; in the late stage of germination, various secondary metabolic pathways and signal transduction were also enriched.

### 2.6. Kyoto Encyclopedia of Genes and Genomes (KEGG) Analysis of the DEGs

**Figure 6:**
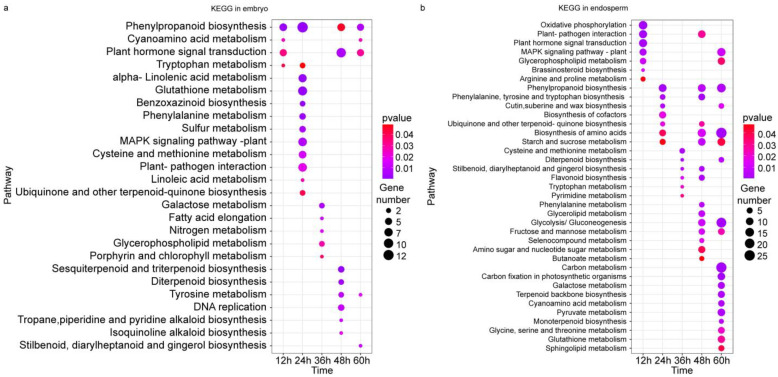
Kyoto Encyclopedia of Genes and Genomes (KEGG) of transcriptional differences during the germination of maize seeds treated with exogenous methionine. (**a**): Top 10 enrichment pathways from different time points results in the embryo; (**b**): top 10 enrichment pathways from different time points results in the endosperm. The bubble color indicates the *p* value. The size of the dots indicates how many differential genes are present in each pathway.

KEGG analysis shows multiple metabolisms to be enriched upon methionine treatment. Several enrichments were repeated in both the embryo and endosperm, such as phenylpropanoid biosynthesis (ko00940); phenylalanine metabolism (ko00360); tryptophan metabolism (ko00380); plant hormone signal transduction (ko04075); MAPK signaling pathway (ko04016); glutathione metabolism (ko00480); diterpenoid biosynthesis (ko00904); stilbenoid, diarylheptanoid, and gingerol biosynthesis (ko00945); and plant–pathogen interaction (ko04626). In the embryo, the repeated enrichments of phenylpropanoid biosynthesis and tryptophan metabolism both appeared at 12 and 24 h, which was consistent with the results of GO analysis, and more pathways were enriched at 24 h (Figure 6a). Compared with the embryo, more metabolisms and more genes were enriched at 48 and 60 h in the endosperm. Phenylpropanoid biosynthesis, the biosynthesis of amino acids (ko01230), starch and sucrose metabolism (ko00500), fructose and mannose metabolism (ko00051), and glycolysis (ko00010) were repeatedly enriched at 48 and 60 h. Additionally, a broader range of metabolic pathways, including carbon metabolism (ko01200); carbon fixation in photosynthetic organisms (ko00710); galactose metabolism (ko00052); terpenoid backbone biosynthesis (ko00900); cyanoamino acid metabolism (ko00460); pyruvate metabolism (ko00620); monoterpenoid biosynthesis (ko00902); glycine, serine and threonine metabolism (ko00260); glutathione metabolism (ko00480); and sphingolipid metabolism (ko00600) were enriched at 60 h (Figure 6b).

### 2.7. Validation of DEGs by Quantitative Real-Time PCR (qRT-PCR)

**Figure 7:**
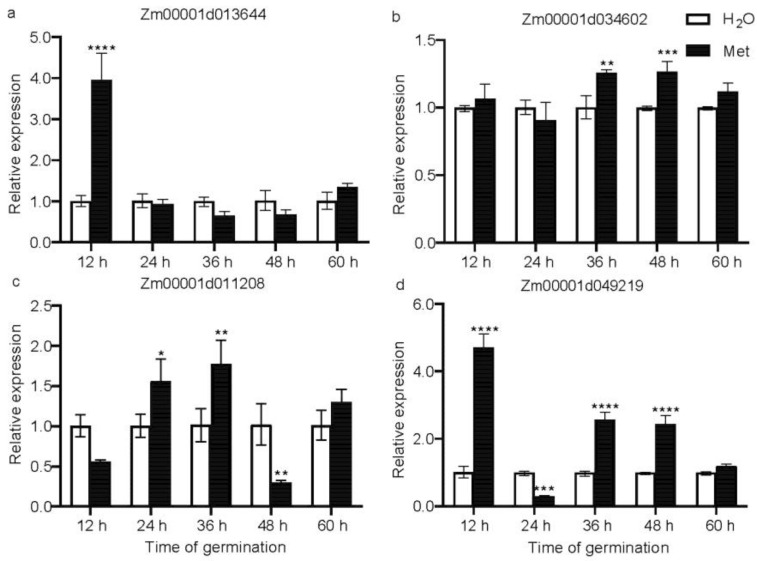
qRT-qPCR verification of four deferentially expressed genes. (**a**): Zm00001d013644; (**b**): Zm00001d034602; (**c**): Zm00001d011208; (**d**): Zm00001d049219. *, *p* < 0.05; **, *p* < 0.01; ***, *p* < 0.001; ****, *p* < 0.0001.

To ensure the reliability of the RNA-seq results, genes that were differentially expressed during the germination process upon methionine treatment were selected for qRT-PCR validation (Figure 7). Among these differential genes in the endosperm, *METHIONINE SYNTHASE 2* (*METS2*, *Zm00001d013644*, Figure 7a), and *5,10-METHYLENETETRAHYDROFOLATE REDUCTASE* (*MTHFR*, *Zm00001d034602*, Figure 7b) were found to be related to methionine and folate synthesis [24], and the qRT-PCR results were similar to the transcriptome results. Compared to the water treatment, the transcriptional levels of *METS2* significantly increased after 12 h of methionine treatment. The transcriptional levels of *MTHFR* increased during the middle and late stages of germination, which have been related to the changes in the content of methionine and 5-M-THF observed during the germination process (Figure 2). Additionally, an important gene involved in the biosynthesis of ethylene was also observed to increase significantly under methionine treatment. The transcript levels of *Zm00001d011208*, which encodes the last biosynthetic step of ethylene, 1-aminocyclopropane-1-carboxylate oxidase 5 (ACCO5) [25], increased significantly at 24 and 36 h (Figure 7c). Increased transcript levels of *Zm00001d 049219*, encoding cysteine protease 17 (CCP17) in relation to protein hydrolases during germination [26], in the embryo were also observed (Figure 7d). The expression patterns of these DEGs closely match the results from the RNA-seq analysis, providing additional evidence of the data’s reproducibility.

### 2.8. Multiple Phenylpropanoid Biosynthetic Processes Were Mainly Activated by the Application of Methionine

**Figure 8:**
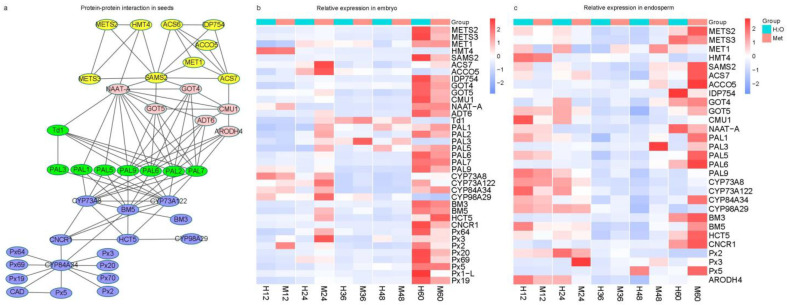
The application of methionine has an impact on genes involved in phenylalanine, tyrosine, tryptophan, and phenylpropanoid biosynthetic processes during maize seed germination. (**a**): Protein–protein interaction network of methionine and cysteine metabolism; phenylalanine, tyrosine, tryptophan, and phenylpropanoid biosynthetic processes. Yellow, genes involved in methionine and cysteine metabolism; pink, genes involved in phenylalanine, tyrosine and tryptophan biosynthesis; green, phenylalanine metabolism and phenylpropanoid biosynthesis; blue, phenylpropanoid biosynthesis; (**b**): the relative expression levels of genes involved in methionine and cysteine metabolism; phenylalanine, tyrosine, tryptophan, and phenylpropanoid biosynthetic processes in the embryo at different time points during germination; (**c**): the relative expression levels of genes involved methionine and cysteine metabolism; and phenylalanine, tyrosine, tryptophan, and phenylpropanoid biosynthetic processes in the endosperm at different time points during germination. Protein annotations and abbreviations are in Table S4. Molecules: H_2_O, water, H; Met, methionine, M.

The phenylpropanoid biosynthetic process was repeatedly enriched at 12 and 24 h in the embryo and at 48 and 60 h in the endosperm (Figure 4, Figure 5 and Figure 6), which indicates that the phenylpropanoid biosynthetic process can be significantly influenced by the application of methionine during different stages of germination. Thus, protein–protein interaction (PPI) network analysis was preformed using DEGs from both the embryo and endosperm by STRING [27], and the PPI network among these pathways was screened to identify the connection between methionine/cysteine metabolism and phenylpropanoid biosynthesis (Figure 8a). Interestingly most of them were connected at four levels: (1) genes involved in methionine and cysteine metabolism—these genes were connected with phenylalanine, tyrosine, tryptophan biosynthetic processes by *HOMOCYSTEINE S-METHYLTRANSFERASE 4* (*HMT4*, *Zm00001d043302*), *SAMS2* (*Zm00001d009146*), and *1-AMINOCYCLOPROPANE-1-CARBOXYLATE SYNTHASE 7* (*ACS7*, *Zm00001d026060*); (2) genes involved in phenylalanine, tyrosine and tryptophan biosynthesis—these genes were connected with phenylalanine metabolism by *NICOTIANAMINE AMINOTRANSFERASE* (*NAAT-A*, *Zm00001d048736*), *ASPARTATE AMINOTRANSFERASE 5* (*GOT5*, *Zm00001d010190*), *AROGENATE DEHYDRATASE* 6 (*ADT6*, *Zm00001d003830*), and *AROGENATE DEHYDROGENASE 4* (*ARODH4*, *Zm00001d046168*); (3) genes involved in phenylalanine metabolism—*PHENYLALANINE AMMONIA LYASE* (*Zm00001d051164*, *PAL1*; *Zm00001d003016*, *PAL2*; *Zm00001d051161*, *PAL3*; *Zm00001d051163*, *PAL5*; *Zm00001d017279*, *PAL7*; *Zm00001d017275*, *PAL9*) connected phenylalanine metabolism with *TRANS-CINNAMATE 4-MONOOXYGENASE CYP73A8* (*Zm00001d016471*), *TRANS-CINNAMATE 4-MONOOXYGENASE CYP73A122* (*Zm00001d037849*), and *4-COUMARATE—COA LIGASE* (*BM5*, *Zm00001d015459*) in phenylpropanoid biosynthetic process; (4) genes involved in the phenylpropanoid biosynthesis—multiple interaction levels involved in phenylpropanoid biosynthetic process were found, such as *TRANS-CINNAMATE 4-MONOOXYGENASE* (*Zm00001d037849*, *CYP73A122*; *Zm00001d016471*, *CYP73A8*; *Zm00001d013862*, *CYP84A34*; *Zm00001d038555*, *CYP98A29*), *PEROXIDASE* (*Zm00001d040705*, *Px19*; *Zm00001d038598*, *Px2*; *Zm00001d032405*, *Px20*; *Zm00001d022457*, *Px3*; *Zm00001d037550*, *Px5*; *Zm00001d009138*, *Px64*; *Zm00001d037410*, *Px69*; *Zm00001d029604*, *Px70*), BM3 (Zm00001d049541), and *4-COUMARATE--COA LIGASE*(Zm00001d015459, BM5; Figure 8a; Table S4). The relative expressions of these genes indicate that, at 24 h in the embryo, most of them are triggered by the application of methionine, and at 60 h, most of them decrease (Figure 8b). In the endosperm, the relative expression of most genes at 12 and 24 h was inhibited, but they started to become active from 48 h and increased more at 60 h due to the application of methionine (Figure 8c). These results indicate that the application of methionine increases the germination rate of maize seeds by triggering multiple phenylpropanoid biosynthetic genes at the transcript level.

## 3. Discussion

Corn is a vital economic crop in China and an indispensable feed material in animal husbandry. As the starting point for plant growth and development, seeds not only affect growth but also affect the quality and yield of corn. The germination of seeds not only requires suitable external conditions, but also relies on their own nutritional supplies of storage substances, among which starches, proteins, and sugars are closely related to seed germination [2]. Storage proteins are hydrolyzed into free amino acids under the action of proteases, and, together with the de novo biosynthesis of amino acids, these processes provide nutrients for growing seeds [26]. During the early stage of seed germination, some substances can be added to improve the uniformity of the seeds, thereby breaking dormancy, improving the germination rate in *Arabidopsis*, and these include methionine [20]. Methionine is an essential amino acid and an important metabolite involved in protein synthesis. It is also a precursor of methyl donor SAM, plant hormone ethylene, and polyamines [28]. An increased germination rate was also observed in the overexpression of a Met/SAM feedback-insensitive form of the cystathionine γ-synthase gene (*AtD-CGS*) with increased methionine content in the seeds, but the regulatory mechanism of methionine in germination urgently needs to be elucidated [29].

In this study, it was observed that the methionine contents increased in both the embryo and endosperm during germination (Figure 2). Similar to what was observed in *Arabidopsis* [20], exogenous application of methionine in maize resulted in higher free methionine content in the embryo and endosperm than in the control group at 36 h of germination. However, the trends of water or methionine treatment in the embryo and endosperm exhibited little difference. In maize embryo, the methionine content reached a peak at 48 h, which resembled the observation made in *Arabidopsis* seeds [22], and the application of methionine brought this event forward by 12 h. In the endosperm, the methionine contents increased rapidly at 48 h, and the application of methionine also brought the rapid increase forward by 12 h (Figure 2). These phenomena coincided with an increased germination rate at 48 h upon the methionine treatment (Figure 1), indicating that seed germination utilized methionine, and more methionine promotes an accelerated seed germination process. At the early stage in the embryo and the late stage in the endosperm, DEGs were mainly involved in phenylalanine metabolism and phenylpropanoid biosynthesis pathways (Figure 4, Figure 5 and Figure 6). To obtain information about how methionine metabolism is connected with the phenylpropanoid biosynthesis process, the PPI network among these enriched pathways was screened (Figure 8) [27]. Several biosynthetic key genes were identified and connected at different levels in the PPI network results (Figure 8). Among them, some genes involved in seed germination had been reported previously. For example, METS [20] and ACS [25] are involved in methionine and cysteine metabolism; ADT is involved in phenylalanine biosynthesis [18]; PAL is a rate-limiting enzyme in phenylpropanoid biosynthesis [19]; peroxidase catalyzes oxidoreduction to produce ROS during phenylpropanoid biosynthesis [30]. The activation of their expression at the early stages (12 to 24 h) in the embryo and at the late stages (48 to 60 h) upon treatment with methionine (Figure 8b) were positively related to the higher germination rate in seeds (Figure 1). Compared with methionine application in *Arabidopsis* germination, our results further provide insight into how methionine application increases the germination rates of maize by triggering phenylpropanoid biosynthesis.

Meanwhile, other pathways related to the cell structure and cell cycle, which contribute to promoting seed germination, were also enriched in our analysis (Figure 4, Figure 5 and Figure 6). In plant growth and development, cell cycles such as mitosis and cytoplasmic division play important roles, while the cytoskeleton, such as microtubules, plays an important role in plant cell activity [31], indicating that the application of methionine may affect the growth, development, and division differentiation of embryonic cells. After 24 h, the main pathways involved were glycosyltransferase and amylase activity related to hormones such as jasmonic acid, cytokinin, and auxin and most of them were upregulated (Figure 4, Figure 5 and Figure 6), indicating that the application of methionine could affect the response of embryonic cells to plant hormones and the degradation of storage substances such as starches and sugars. At 12 h in the endosperm, pathways such as those involving endopeptidases and glycosyl compound hydrolases were enriched and mostly upregulated, indicating that the addition of methionine accelerates the utilization of sugars and proteins in the endosperm. At 24 to 48 h, various hormone pathways and hydrolases activity were enriched. For example, the upregulation of *ACCO5* was observed to be induced upon the methionine treatment at 24 and 36 h (Figure 7c). It has been reported that ethylene promotes seed germination in both *Arabidopsis* and other species [32,33]. Thus, we speculate that ethylene could be involved in improving germination via the application of methionine. Several genes involved in proteolysis were also found to be induced, such as *CCP17* (Figure 7d). It was reported that maize germination is accompanied by the appearance of cysteine protease(s) [34]. Thus, the application of methionine could have an important impact on the degradation and utilization of storage proteins during seed germination. At 60 h, more metabolic pathways and signal transduction were enriched indicating the preparation of seedling establishment and development (Figure 6). Compared with the changes in the methionine content, 5-M-THF accumulated continuously in the embryo throughout the entire germination process, and there was no obvious change in the folate content in the endosperm, which was similar to that in pea seeds [24]. The pattern of change was not altered upon methionine treatment, indicating that the improved germination was mainly triggered by methionine.

## 4. Materials and Methods

### 4.1. Plant Materials and Growth Conditions

*Zea mays* (maize) inbred line B73 was used in the seed germination experiment. Seed germination and treatment was preformed according to Zhang et al. with minor modifications [35]. Seeds were soaked in a 2% sodium hypochlorite solution for 10 min to disinfect, rinsed thoroughly with running water, and then rinsed with sterile water once. The filter paper was put into Petri dishes. A total of 50 corn seeds were put on the filter paper, and there were 10 dishes for the germination experiment. Next, 5 dishes were added with 30 mL sterile water as the control group, and another 5 were added with 30 mL methionine solution as the treatment group. For this experiment, 25, 50, and 75 μM methionine was used before and 75 μM methionine appeared to have the best effect in increasing seed germination rates and was chosen in this study. All Petri dishes were placed into a plant constant temperature incubator at 28 ± 1 centigrade (°C) in the dark. Photos were taken every 12 h to record the germination situation and the photo of 60 h was shown as the germination results. Germination was considered to have occurred if the emerged radicle exceeded 1mm in length. The germination rate was calculated as the percentage of germinated seeds at each time point. All assays were replicated at least five times to minimize experimental error. Maize embryos and endosperm at 12, 24, 36, 48, and 60 h were separated and frozen for the following experiments.

### 4.2. Measurement of Free Methionine Contents by Liquid Chromatography Mass Spectrometer (LC/MS)

The free methionine contents were measured according to Nikiforova et al. with minor modifications [36]. A total of 100 mg of grain powder was weighted and placed in a 1.5 mL Eppendorf tube. Next, 300 μL ice-cold 80% methanol aqueous solution and 100 μL dithiothreitol (DTT; 15 mg/mL) solution was added in the tube. The samples were vortexed and mixed for 1 min (min), and placed on ice for 30 min, followed by centrifuging them at 12,000 rpm at 4 °C for 10 min. After that, the supernatant was transferred in a new Eppendorf tube. Then, 300 μL ice-cold isopropanol and 100 μL DTT (15 mg/mL) solution was added to the sediment, and the same procedure was repeated. The supernatant was added in the same Eppendorf tube in the earlier step. A total of 100 μL of the mixed supernatant was filtered through 0.22 μM nylon membrane into a brown bottle tube for LC/MS detection. The filtered solution was separated on a ACQUITY UPLC HSS T3 column (2.1 × 100 mm, 5 μm; Waters, Taunton, MA, USA, www.waters.com) using an HPLC instrument (Waters, Taunton, MA, USA, www.waters.com) equipped with a 5500 QTRAP mass spectrometer together with electrospray ionization (ESI) source (Applied Biosystems SCIEX, Shanghai, China, www.sciex.com.cn). Elution was performed using a binary gradient of acetonitrile: formic acid (1000:1; mobile phase A) and formic acid aqueous solution (containing 0.1% formic acid; mobile phase B), according to the following program: 0.0–1.0 min, 97% A/3% B; 1.1–6.1 min, 80% A/20% B; 6.2–13.0 min, 97% A/3% B. The flow rate was 0.3 mL/min and the column temperature was 4 °C. Analyte peak areas were normalized to standard peak areas and converted to nmol/L using the standard curve. The column temperature was maintained at 4 °C. The injection volume was 20 µL for each experiment. A Waters triple quadrupole tandem MS (Waters USA, www.waters.com) coupled with an ESI interface was used for mass analyses and quantification of target analytes. The mass spectrometer was operated in positive-ion mode. The parameters were optimized for target analytes with a gas temperature of 500 °C, nebulizer pressure of 50 psi, and capillary voltage of 5500 V. The parameters for standards were *m*/*z* 150–56.2, 16 eV for methionine. System operation, data acquisition, and data analyses were performed with Analyst 1.6.3 Quantitative Processing Software (Applied 161 Biosystems, Shanghai, China, accessed on 4 June 2021) and Microsoft Excel software (Microsoft 365, Seattle, WA, USA, accessed on 1 September 2019). Three biological replicates were prepared for each sample.

### 4.3. Measurement of 5-M-THF Contents by LC/MS

The 5-M-THF measurements were performed as previously described [37]. Briefly, embryos and endosperms per replicate were ground into a fine powder under liquid nitrogen. Thereafter, 50 mg of the fine kernel powder was mixed with 1 mL of freshly prepared extraction buffer (50 mM phosphate buffer [pH 7.0], 0.5% sodium ascorbate, and 0.2% β-mercaptoethanol) mixed with methotrexate as an internal standard. Then, the mixture was boiled for 10 min in a water bath and cooled on ice for 10 min. Subsequently, 30 μL of rat serum was added to each sample, which was incubated for 4 h at 37 °C to deconjugate the polyglutamylated tails. Next, the samples were boiled for 10 min, cooled on ice for 10 min, and centrifuged at 13,000 rpm and 4 °C for 10 min. The supernatants (400 μL) were transferred into 3-kDa ultra-filtration tubes (Millipore, Burlington, MA, USA) and centrifuged at 13,000 rpm and 4 °C for 20 min. The resulting solution was used for folate analysis by LC/MS. Three biological replicates were prepared for each sample.

### 4.4. Transcriptome Sequencing Data Analysis

The embryos and endosperms of B73 maize seeds treated with methionine or water at 0, 12, 24, 36, 48, and 60 h of germination were used for RNA extraction, and three biological replicates were set up. Transcriptome sequencing was completed by Beijing Qingke Biotechnology Co., Ltd. (Beijing, China). A total of 66 samples were tested using the Illumina platform (San Diego, CA, USA. www.illumina.com), producing 640.20 Gb of data with a Q30 base percentage of 88.38% or higher. Gene differential analysis was standardized using R-based DESeq2 software (r-project.org, accessed on 1 July 2022), and then the expression multiple (log2FoldChange) and *p*-value values of the compared samples were calculated to reflect the magnitude of differences between the samples.

Based on the FPKM value, differential genes were screened and compared between methionine and water treatments in maize embryos or endosperm during the same time point. The gene IDs of DEGs were converted into the corresponding Entrez ID through the v4 version of the gene model cross reference on the maizeGDB website as the subsequent input file, then, they were used in the online websites KOBAS 3.0 (http://kobas.cbi.pku.edu.cn/, accessed on 5 December 2022) and Agrigo v2 (http://systemsbiology.cau.edu.cn/agriGOv2/, accessed on 5 December 2022) to enrich and analyze the differential genes. The enrichment results obtained on these two online websites determine the pathway that needs attention based on *p* value < 0.05. This result can be visualized through R and SRplot (www.bioinformatics.com.cn, accessed on 5 December 2022).

### 4.5. Quantitative Real-Time PCR (qRT-PCR)

The same 2.0 μg RNA used for transcriptome analysis was used to synthesize first-strand cDNA using M-MLV reverse transcriptase, and qRT-PCR analyses were conducted using TransStart Top Green qPCR SuperMix (TRANS, Beijing, China) on an ABI 7500 thermocycler (Applied Biosystems, Marsiling industrial, Singapore). The maize GAPDH gene was provided as an internal standard. The primer sequences are listed in Table S5. A 2^–ΔΔCt^-based calculation was used to quantify gene expression.

### 4.6. Protein–Protein Interaction (PPI) Networks Analysis

The Protein–protein interaction (PPI) networks analysis was predicted using DEGs from both the embryo and endosperm with STRING (version 11.0; https://string-db.org/cgi/input.pl, accessed on 5 July 2023) [27]. The full network type was used to complete the analysis, and the edges represent the physical and functional protein associations. The minimum interaction score required was set to intermediate confidence (0.400).

## 5. Conclusions

Exogenous application of methionine increased seed germination rate, leading to differences in the content of seed methionine at 36 h, which brought the rapid increase forward by 12 h in the embryo and endosperm. Significant changes in the transcription levels of genes involved in metabolic processes, such as those associated with the cell structure and cell cycle, hormones, and amylase in the embryo, were observed. Furthermore, substantial changes were observed in genes associated with endosperm hormones, secondary metabolites, and endopeptidases, which are implicated in metabolites biosynthesis and degradation during germination. These findings suggest that methionine may primarily influence cell proliferation and differentiation in the embryo, consequently affecting the degradation of storage substances and signal transduction in the endosperm. PPI network analysis also predicted the activation of multiple phenylpropanoid biosynthetic genes, such as genes encoding phenylalanine ammonia lyase, trans-cinnamate 4-monooxygenas, and peroxidase at the transcript level, indicating that multiple phenylpropanoid biosynthetic genes were triggered upon the methionine treatment during germination. These findings establish a theoretical foundation for the further promotion of maize seed germination and serve as a valuable theoretical resource for seed priming strategies.

## Data Availability

Data are contained within the article and Supplementary Materials.

## Supplementary Materials

The following supporting information can be downloaded at: https://www.mdpi.com/article/10.3390/plants12223802/s1, Table S1: Overview of transcriptome sequencing in maize embryos upon the water (H) and methionine (M) treatment during germination. Table S2: Overview of transcriptome sequencing in maize endosperms upon the water (H) and methionine (M) treatment during germination. Table S3: Annotation results using various databases; Table S4: Protein annotations and abbreviations involved in Figure 8; Table S5: Primer sequences in this study.

## Abbreviations

5-M-THF	5-methyl-tetrahydrofolate
ABA	Abscisic acid
ABI	Abscisic-acid-insensitive transcription factor
ACCO	1-aminocyclopropane-1-carboxylate oxidase
ACS	1-aminocyclopropane-1-carboxylate synthase
ADT	Arogenate dehydratase
ARODH	Arogenate dehydrogenase
BM3	Caffeic acid 3-O-methyltransferase
BM5	4-coumarate--CoA ligase
CCP	Cysteine protease
CYP	Trans-cinnamate 4-monooxygenas
DEGs	Differentially expressed genes
DTT	Dithiothreitol
GA	Gibberellins
GID1	*Gibberellin-intensive DWARF1*
GO	Gene Ontology
GOT5	Aspartate aminotransferase
h	Hours
H	H_2_O, water
HMT4	Homocysteine S-methyltransferase
JA	Jasmonic acid
JAZ	JASMONATE ZIM DOMAIN
KEGG	Kyoto Encyclopedia of Genes and Genomes
LC/MS	Liquid Chromatography Mass Spectrometer
M	Met, Methionine
min	Minutes
METS	Methionine synthase
MTHFR	5,10-methylenetetrahydrofolate reductase
MYB	V-myb avian myeloplastosis viral oncogene homolog
NAAT-A	Nicotianamine aminotransferase
PAL	Phenylalanine ammonia lyase
PPI	Protein–protein interaction
qRT-PCR	Quantitative real-time PCR
RGL2	RGA-LIKE 2
ROS	Reactive oxygen species
SAM	S-adenosylmethionine
SAMS	S-adenosylmethionine synthetase
SD1	SEMIDWARF1
SnRK2	Sucrose non-fermenting-1-related protein kinase 2s
