# Supplementary material for: Application of Methionine Increases the Germination Rate of Maize Seeds by Triggering Multiple Phenylpropanoid Biosynthetic Genes at Transcript Levels
Source: Plants (Basel). 2023 Nov 8;12(22):3802. doi: 10.3390/plants12223802 (PMC10675280; doi:10.3390/plants12223802)
Supplement: Supplementary file 1 [file plants-12-03802-s001.zip › plants-2668450-supplementary.pdf]

Table S1. Overview of transcriptome sequencing in maize embryos upon the water (H) and methionine (M) treatment during germination.

Embryo	sample ID	rep	clean reads	Q20 bases (%)	Q30 bases (%)	GC content (%)	mapping reads	mapped reads with unique loci	mapping ratio
H00	H01	rep1	59707350	96.29%	89.53%	56.17%	56987893	52701759	95.45%
	H02	rep2	51401268	95.91%	88.38%	54.29%	48944449	45151809	95.22%
	H03	rep3	50545422	95.90%	88.39%	55.79%	48157113	45069923	95.27%
H12	H121	rep1	47441258	96.02%	88.72%	55.41%	45213692	42697080	95.30%
	H122	rep2	49830018	96.08%	88.85%	53.39%	47586609	44691138	95.50%
	H123	rep3	52120110	96.14%	89.05%	54.88%	49658450	45883407	95.28%
H24	H241	rep1	67128548	96.45%	89.89%	53.19%	64250850	61431073	95.71%
	H242	rep2	51286600	96.44%	89.89%	53.46%	49094509	47010135	95.73%
	H243	rep3	52720000	96.18%	89.01%	53.78%	50502368	48517289	95.79%
H36	H361	rep1	60313026	97.14%	91.16%	53.75%	58314522	55428234	96.69%
	H362	rep2	56199630	96.51%	90.12%	53.34%	53697824	51246328	95.55%
	H363	rep3	50195768	96.48%	90.00%	54.45%	48087832	45358822	95.80%
H48	H481	rep1	50206974	96.26%	89.40%	55.10%	47970816	44443846	95.55%
	H482	rep2	53184838	96.17%	89.06%	54.35%	50855563	48126195	95.62%
	H483	rep3	40601648	96.10%	88.89%	54.28%	38835442	36436139	95.65%
H60	H601	rep1	48764330	96.64%	90.31%	53.75%	46878434	45055712	96.13%
	H602	rep2	69753182	96.11%	88.87%	53.72%	66750911	64426579	95.70%
	H603	rep3	56763060	96.39%	89.77%	53.98%	54358843	52486144	95.76%
M12	M121	rep1	52510524	96.30%	89.41%	55.12%	50177697	47430039	95.56%
	M122	rep2	52433336	96.28%	89.39%	54.58%	50151671	46058052	95.65%
	M123	rep3	44545088	96.33%	89.49%	53.93%	42657287	40211187	95.76%
M24	M241	rep1	69926650	98.70%	95.58%	53.86%	68496317	65036749	97.95%
	M242	rep2	40998154	96.17%	89.11%	54.45%	39037872	37190614	95.22%
	M243	rep3	40690522	96.10%	88.80%	53.51%	38930162	37396035	95.67%
M36	M361	rep1	59795262	96.55%	90.18%	53.46%	57293004	54760093	95.82%
	M362	rep2	61069778	96.47%	89.96%	53.84%	58436295	55473799	95.69%
	M363	rep3	43430300	96.22%	89.12%	53.94%	41593480	39716555	95.77%
M48	M481	rep1	68878314	96.11%	88.88%	53.58%	65956285	61149591	95.76%
	M482	rep2	66401512	96.73%	90.77%	53.44%	63490396	60119900	95.62%
	M483	rep3	65237010	96.59%	90.31%	54.31%	62526223	58254397	95.84%
M60	M601	rep1	46840704	96.41%	89.76%	53.88%	44812482	42787537	95.67%
	M602	rep2	55294006	96.22%	89.20%	53.94%	52906934	51268098	95.68%
	M603	rep3	40238474	96.12%	88.92%	55.08%	38447160	36651033	95.55%

Notes: 00, 0 hour; 12, 12 hours; 24, 24 hours; 36, 36 hours; 48, 48 hours; 60, 60 hours.

Table S2. Overview of transcriptome sequencing in maize endosperms upon the water (H) and methionine (M) treatment during germination.

Endosperm	sample ID	rep	clean reads	Q20 bases (%)	Q30 bases (%)	GC content (%)	mapping reads	mapped reads with unique loci	mapping ratio
RH00	RH01	rep1	72246318	98.24%	94.38%	60.51%	70733522	59910653	97.91%
	RH02	rep2	75734520	98.18%	94.11%	60.68%	74185757	69728175	97.96%
	RH03	rep3	73641826	98.21%	94.22%	59.96%	72109851	66191701	97.92%
RH12	RH121	rep1	69956048	98.09%	93.95%	61.70%	68513166	63910298	97.94%
	RH122	rep2	58482098	98.19%	94.10%	57.94%	57002955	53424741	97.47%
	RH123	rep3	65079790	98.31%	94.45%	59.02%	63685211	60755066	97.86%
RH24	RH241	rep1	89928532	98.48%	94.87%	59.70%	88517089	85570802	98.43%
	RH242	rep2	74820196	95.86%	90.40%	58.22%	71502450	69382290	95.57%
	RH243	rep3	85341072	98.54%	95.03%	59.89%	83873327	80329611	98.28%
RH36	RH361	rep1	78353544	98.54%	95.03%	59.39%	77083067	74196333	98.38%
	RH362	rep2	85435822	98.44%	94.71%	60.16%	84111188	81075014	98.45%
	RH363	rep3	87015072	98.55%	95.08%	60.09%	85382906	81605989	98.12%
RH48	RH481	rep1	92440744	98.40%	94.68%	61.46%	90845117	87561983	98.27%
	RH482	rep2	78520804	98.46%	94.77%	59.63%	77265023	74136789	98.40%
	RH483	rep3	76783620	98.53%	95.07%	60.26%	75577725	72492394	98.43%
RH60	RH601	rep1	70230690	98.49%	94.86%	59.18%	69198229	66559031	98.53%
	RH602	rep2	64109216	95.59%	89.98%	59.51%	60674709	58654623	94.64%
	RH603	rep3	65716316	95.75%	90.29%	60.13%	62398927	60705585	94.95%
RM12	RM121	rep1	60911658	98.34%	94.54%	58.46%	59754116	57255918	98.10%
	RM122	rep2	83292556	98.52%	94.99%	59.15%	81615121	76766841	97.99%
	RM123	rep3	88959410	98.46%	94.91%	60.97%	86990613	81964985	97.79%
RM24	RM241	rep1	83007382	98.44%	94.70%	59.31%	81651521	78868973	98.37%
	RM242	rep2	64062894	95.54%	89.71%	58.90%	61140092	59130586	95.44%
	RM243	rep3	94493398	98.52%	94.84%	60.14%	93084919	89879392	98.51%
RM36	RM361	rep1	79721590	98.34%	94.45%	60.95%	78292334	74821942	98.21%
	RM362	rep2	67783552	98.51%	94.89%	59.34%	66137271	63704690	97.57%
	RM363	rep3	94160836	98.35%	94.48%	59.96%	92576831	89465814	98.32%
RM48	RM481	rep1	65503976	95.58%	89.89%	60.46%	62295552	60225502	95.10%
	RM482	rep2	81845372	98.48%	94.91%	59.40%	80539116	77724488	98.40%
	RM483	rep3	95971342	98.48%	94.89%	59.84%	94356959	90421314	98.32%
RM60	RM601	rep1	60513970	95.49%	89.74%	59.07%	57219999	55204928	94.56%
	RM602	rep2	64483098	95.82%	90.45%	60.14%	61229818	59413784	94.95%
	RM603	rep3	66425564	95.59%	89.91%	58.46%	63060750	61159138	94.93%

Notes: 00, 0 hour; 12, 12 hours; 24, 24 hours; 36, 36 hours; 48, 48 hours; 60, 60 hours.

Table S3: Annotation results using various databases.

Database	number
GO（Gene Ontology）	3,441
KEGG	691
KOG（Cluster of Orthologous Groups of proteins）	2,384
Pfam（Protein families database of alignments and hidden Markov models）	2,490
Swiss-Prot	2,534
eggNOG（evolutionary genealogy of genes:Non-supervised Orthologous）	6,023
Non-Redundant Protein Sequence Database	9,732
All	9,890

Table S4. Protein annotations and abbreviations involved in Figure 8.

GeneID	Name	Annotation	KEGG
Zm00001d011208	ACCO5	1-aminocyclopropane-1-carboxylate oxidase	Cysteine and methionine metabolism
Zm00001d033862	ACS6	1-aminocyclopropane-1-carboxylate synthase	Cysteine and methionine metabolism
Zm00001d026060	ACS7	1-aminocyclopropane-1-carboxylate synthase	Cysteine and methionine metabolism
Zm00001d043302	HMT4	Homocysteine S-methyltransferase	Cysteine and methionine metabolism
Zm00001d020544	IDP754	S-adenosylmethionine decarboxylase proenzyme	Cysteine and methionine metabolism
Zm00001d018977	MET1	DNA (cytosine-5)-methyltransferase	Cysteine and methionine metabolism
Zm00001d013644	METS2	5-methyltetrahydropteroyltriglutamate--homocysteine methyltransferase	Cysteine and methionine metabolism
Zm00001d033480	METS3	5-methyltetrahydropteroyltriglutamate--homocysteine methyltransferase	Cysteine and methionine metabolism
Zm00001d009146	SAMS2	S-adenosylmethionine synthetase	Cysteine and methionine metabolism
Zm00001d048736	NAAT-A	Nicotianamine aminotransferase	Cysteine and methionine metabolism / Phenylalanine metabolism / Tyrosine metabolism
Zm00001d003830	ADT6	Arogenate dehydratase	Phenylalanine, tyrosine and tryptophan biosynthesis
Zm00001d046168	ARODH4	Arogenate dehydrogenase	Phenylalanine, tyrosine and tryptophan biosynthesis
Zm00001d012674	CMU1	Chorismate mutase	Phenylalanine, tyrosine and tryptophan biosynthesis
Zm00001d042685	GOT4	Glutamate-oxaloacetate transaminase	Phenylalanine, tyrosine and tryptophan biosynthesis / Tyrosine metabolism / Phenylalanine metabolism (ko00360)
Zm00001d010190	GOT5	Bifunctional aspartate aminotransferase and glutamate/aspartate-prephenate aminotransferase	Phenylalanine, tyrosine and tryptophan biosynthesis / Tyrosine metabolism / Phenylalanine metabolism (ko00360)
Zm00001d024664	Td1	Tyrosine decarboxylase	Phenylalanine metabolism, Tyrosine metabolism, Tryptophan metabolism
Zm00001d051164	PAL1	Phenylalanine ammonia lyase	Phenylalanine metabolism / Phenylpropanoid biosynthesis
Zm00001d003016	PAL2	Phenylalanine ammonia lyase	Phenylalanine metabolism / Phenylpropanoid biosynthesis
Zm00001d051161	PAL3	Phenylalanine ammonia lyase	Phenylalanine metabolism / Phenylpropanoid biosynthesis
Zm00001d051163	PAL5	Phenylalanine ammonia lyase	Phenylalanine metabolism /

			Phenylpropanoid biosynthesis
Zm00001d003015	PAL6	Phenylalanine ammonia lyase	Phenylalanine metabolism / Phenylpropanoid biosynthesis
Zm00001d017279	PAL7	Phenylalanine ammonia lyase	Phenylalanine metabolism / Phenylpropanoid biosynthesis
Zm00001d017275	PAL9	Phenylalanine ammonia lyase	Phenylalanine metabolism / Phenylpropanoid biosynthesis
Zm00001d049541	BM3	Caffeic acid 3-O-methyltransferase	Phenylpropanoid biosynthesis/Tryptophan metabolism
Zm00001d015459	BM5	4-coumarate--CoA ligase	Phenylpropanoid biosynthesis
Zm00001d024314	CAD	Cinnamyl alcohol dehydrogenase	Phenylpropanoid biosynthesis
Zm00001d032152	CNCR1	Cinnamoyl CoA reductase	Phenylpropanoid biosynthesis
Zm00001d037849	CYP73A122	Trans-cinnamate 4-monooxygenase	Phenylpropanoid biosynthesis
Zm00001d016471	CYP73A8	Trans-cinnamate 4-monooxygenase	Phenylpropanoid biosynthesis
Zm00001d013862	CYP84A34	Trans-cinnamate 4-monooxygenase	Phenylpropanoid biosynthesis
Zm00001d038555	CYP98A29	cytochrome P450 superfamily protein	Phenylpropanoid biosynthesis
Zm00001d003129	HCT5	Hydroxycinnamoyltransferase	Phenylpropanoid biosynthesis
Zm00001d040705	Px19	Peroxidase	Phenylpropanoid biosynthesis
Zm00001d038598	Px2	Peroxidase	Phenylpropanoid biosynthesis
Zm00001d032405	Px20	Peroxidase	Phenylpropanoid biosynthesis
Zm00001d022457	Px3	Peroxidase	Phenylpropanoid biosynthesis
Zm00001d037550	Px5	Peroxidase	Phenylpropanoid biosynthesis
Zm00001d009138	Px64	Peroxidase	Phenylpropanoid biosynthesis
Zm00001d037410	Px69	Peroxidase	Phenylpropanoid biosynthesis
Zm00001d029604	Px70	Peroxidase	Phenylpropanoid biosynthesis

Table S5. Primer sequences in this study.

Name	Gene ID	Sequence（5′-3′）
ZmGAPDH-F	Zm00001eb080840	CCCTTCATCACCACGGACTAC
ZmGAPDH-R		AACCTTCTTGGCACCACCCT
METS2-F	Zm00001d013644	TAAGATTTTACTGTTATATAGAAAGATGGC
METS2-R		GGGACAATAAAGTGGTAGTTTGTATC
MTHFR1-F	Zm00001d034602	CACCGAACGCCGTGAATGCT
MTHFR1-R		ACGAGGCTGACCAGGTAGTAGG
ACCO5-F	Zm00001d011208	GGACGAAGTTTCGTTGCTGCT
ACCO5-R		TTGTGTTGTTCGCTGCTGCAT
CCP17-F	Zm00001d049219	TCAGACGCCGACGACAACCA
CCP17-R		GCTGCTCCGATAACGACACCAA
